# Crystal structure of strontium dicobalt iron(III) tris­(orthophosphate): SrCo_2_Fe(PO_4_)_3_

**DOI:** 10.1107/S2056989016011373

**Published:** 2016-07-19

**Authors:** Adam Bouraima, Thomas Makani, Abderrazzak Assani, Mohamed Saadi, Lahcen El Ammari

**Affiliations:** a Laboratoire de Chimie du Solide Appliquée, Faculty of Sciences, Mohammed V University in Rabat, Avenue Ibn Battouta, BP 1014, Rabat, Morocco; b Département de Chimie, Faculté des Sciences, Université des Sciences et Techniques de Masuku, BP 943, Franceville, Gabon

**Keywords:** crystal structure, SrCo_2_Fe(PO_4_)_3_, transition metal phosphate, solid-state reaction synthesis, alluaudite-like structure

## Abstract

The title compound, SrCo_2_Fe(PO_4_)_3_, has been synthesized by a solid-state reaction. It crystallizes with the α-CrPO_4_ type structure. In this structure, all atoms are on special positions of the *Imma* space group, except for two O atoms which are located on general positions. The three-dimensional network in the crystal structure is made up of two types of layers stacked normal to (100). The first layer is built from two edge-sharing CoO_6_ octa­hedra, leading to the formation of Co_2_O_10_ dimers that are connected to two PO_4_ tetra­hedra by a common edge and corners. The second layer results from apex-sharing FeO_6_ octa­hedra and PO_4_ tetra­hedra, which form linear chains alternating with a zigzag chain of Sr^II^ cations. These layers are linked together by common vertices of PO_4_ tetra­hedra and FeO_6_ octa­hedra to form an open three-dimensional framework that delimits two types of channels parallel to [100] and [010] where the Sr^II^ cations are located. Each Sr^II^ cation is surrounded by eight O atoms.

## Abstract 2

The transition metal orthophosphate, SrCo_2_Fe(PO_4_)_3_, crystallizes in an alluaudite-type structure. The chains characterizing the alluaudite structure are built up from edge-sharing [CoO_6_] octa­hedra linked together by PO_4_ tetra­hedra.

## Chemical context   

The phosphate literature includes important works on the structural study of transition metal phosphates. The basic framework is built from tetra­hedrally coordinated phospho­rus linked to transition metals *M* in various environments, such as *M*O_*n*_ (*n* = 4, 5 or 6). The manner in which polyhedra are inter­connected generates important structure types with porous or lamellar set-ups that can exhibit inter­esting physical properties. Accordingly, widespread studies have been devoted to this family of compounds, stimulated by the wide range of potential and commercial applications of these materials. Examples include applications in catalysis, as ion exchangers and in the manufacture of lithium and sodium rechargeable batteries. One particular scientific area in our laboratory is focused on investigating new functional phosphates belonging to the alluaudite (Moore, 1971 ▸) or α-CrPO_4_ (Attfield *et al.*, 1988 ▸) structure types, owing to their potential use as new cathode materials for battery devices (Trad *et al.*, 2010 ▸; Kim *et al.*, 2014 ▸; Huang *et al.*, 2015 ▸).

Our earlier hydro­thermal investigations were undertaken with the *A*_2_O–*M*O–P_2_O_5_ and *M*′O–*M*O–P_2_O_5_ systems (*A* = monovalent cations, *M* and *M*′ = divalent cations) with approximate molar ratios *A*:*M*:*P* = 2:3:3 and *M*′:*M*:P = 1:3:3, which characterize the alluaudite or α-CrPO_4_ phases. Those studies involved the synthesis and structural characterization of new phosphates such as Na_2_Co_2_Fe(PO_4_)_3_ (Bouraima *et al.*, 2015 ▸) and Na_1.67_Zn_1.67_Fe_1.33_(PO_4_)_3_ (Khmiyas *et al.*, 2015 ▸) belonging to the alluaudite-type structure group. In addition, divalent and trivalent transition-metal-based phosphates, such as SrNi_2_Fe(PO_4_)_3_ (Ouaatta *et al.*, 2015 ▸) and *M*Mn^II^_2_Mn^III^(PO_4_)_3_ (*M* = Pb, Sr, Ba) (Alhakmi *et al.*, 2013*a* ▸,**b* ▸;* Assani *et al.*, 2013 ▸) have been shown to adopt the α-CrPO_4_ structure type.

In search of a new promising phosphate, a solid-state chemistry investigation of *A*_2_O–*M*O–*M*′_2_O_3_–P_2_O_5_ systems was undertaken. The present work reports on synthesis and crystal structure of the new strontium cobalt iron phosphate, SrCo_2_Fe(PO_4_)_3_, which has the α-CrPO_4_ type structure.

## Structural commentary   

**Figure 1:**
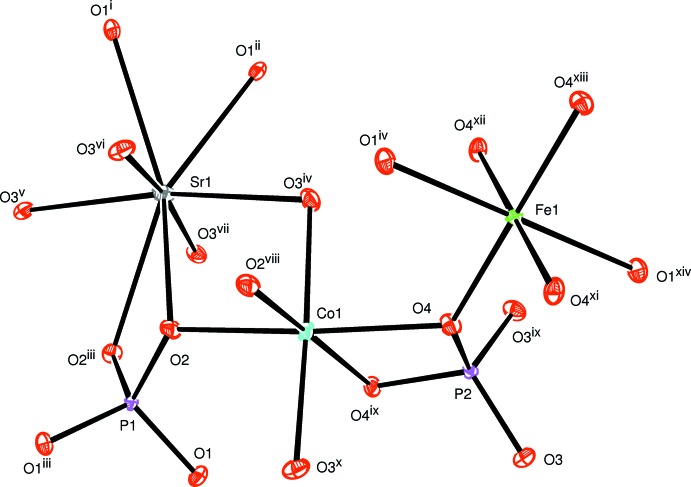
The principal building units in the structure of the title compound. Displacement ellipsoids are drawn at the 50% probability level. [Symmetry codes: (i) −*x* + 2, −*y* + 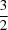, *z* + 1; (ii) *x*, *y*, *z* + 1; (iii) −*x* + 2, −*y* + 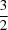, *z*; (iv) −*x* + 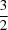, −*y* + 1, *z* + 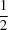; (v) *x* + 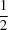, *y* + 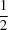, *z* + 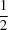; (vi) −*x* + 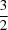, *y* + 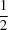, *z* + 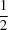; (ix) −*x* + 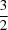, *y*, −*z* + 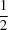; (*x*) *x*, −*y* + 1, −*z*; (xi) −*x* + 1, *y*, *z*; (xii) *x*, −*y* + 1, −*z* + 1; (xiii) −*x* + 1, −*y* + 1, −*z* + 1;(xiv) *x* − 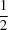, *y*, −*z* + 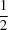.]

**Figure 2:**
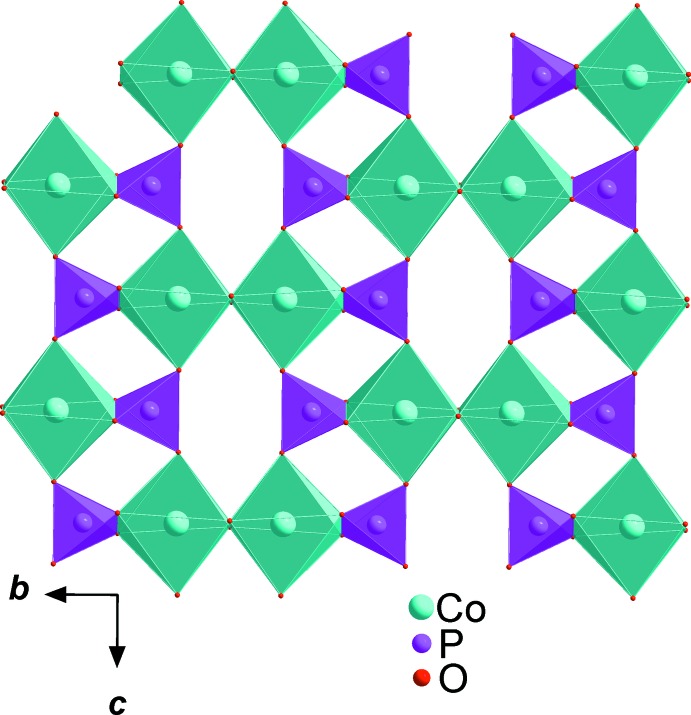
Edge-sharing [CoO_6_] octa­hedra forming a layer parallel to (100).

**Figure 3:**
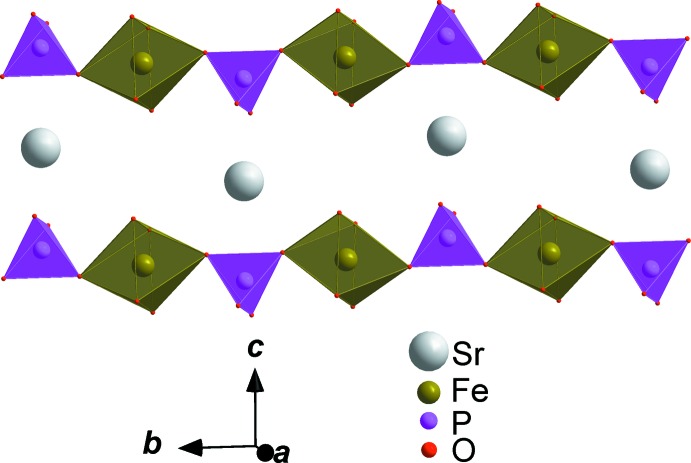
A view along the *a* axis, showing a layer resulting from chains connected *via* vertices of PO_4_ tetra­hedra and FeO_6_ octa­hedra, alternating with a zigzag chain of Sr atoms.

**Figure 4:**
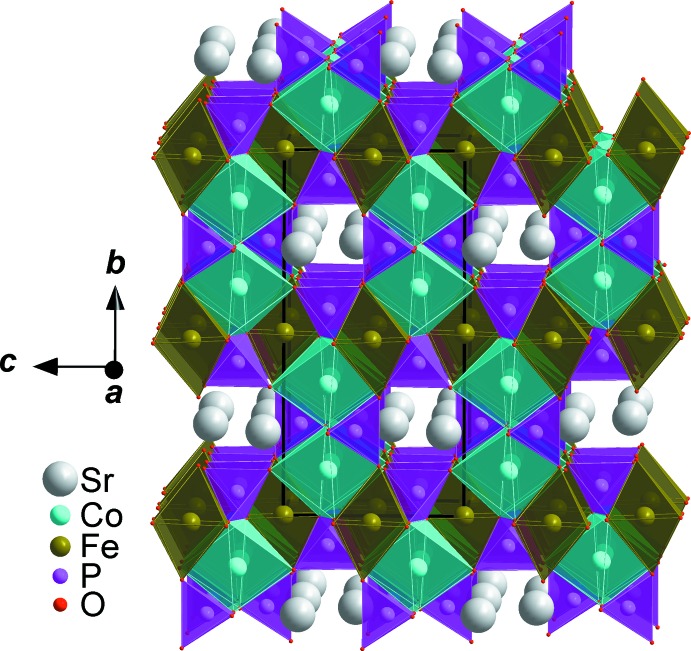
Polyhedral representation of SrCo_2_Fe(PO_4_)_3_, showing channels running along [100].

**Figure 5:**
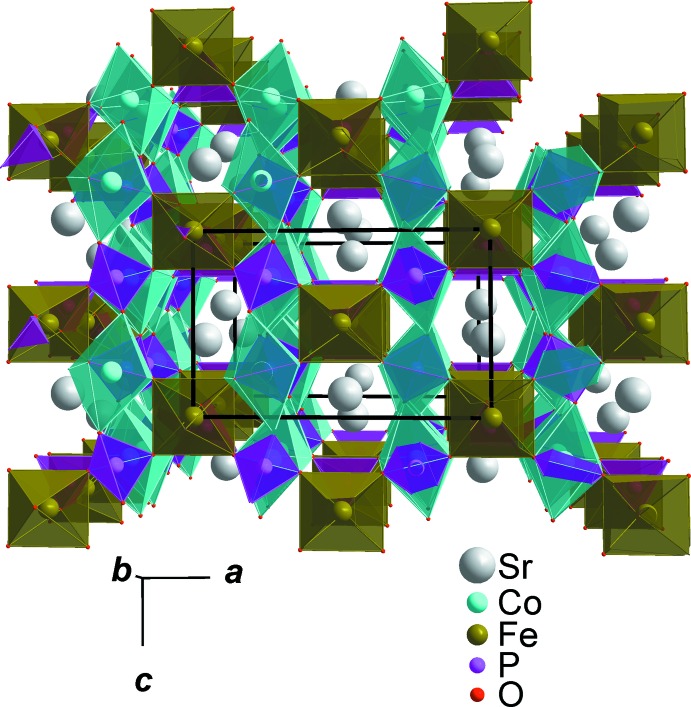
Polyhedral representation of SrCo_2_Fe(PO_4_)_3_, showing channels running along [010].

In the title phosphate, SrCo_2_Fe(PO_4_)_3_, all atoms are on special positions, except two oxygen atoms (O3, O4) which are on general positions of the *Imma* space group. Its three-dimensional structure is constructed on the basis of PO_4_ tetra­hedra, FeO_6_ and CoO_6_ octa­hedra, as shown in Fig. 1 ▸. The connection between these polyhedra produces two types of layers stacked normal to (100). The first layer is built from two edge-sharing CoO_6_ octa­hedra, leading to the formation of Co_2_O_10_ dimers, which are connected to two PO_4_ tetra­hedra by a common edge and vertex, as shown in Fig. 2 ▸. The second layer is formed by alternating FeO_6_ octa­hedra and PO_4_ tetra­hedra, which share corners, building linear chains that surround a zigzag chain of Sr^II^ cations (see Fig. 3 ▸). The layers are joined by the apices of PO_4_ tetra­hedra and FeO_6_ octa­hedra, giving rise to an open three-dimensional framework that delimits two types of channels parallel to [100] and [010] where the Sr^II^ cations are located, as shown in Fig. 4 ▸ and Fig. 5 ▸. This structure is characterized by a stoichiometric composition in which the Sr atom is surrounded by eight oxygen atoms with Sr—O bond lengths that vary between 2.6561 (13) and 2.6690 (9)Å. The same Sr environment is observed in the manganese homologue phosphates, namely *M*Mn^II^_2_Mn^III^(PO_4_)_3_ (*M* = Pb, Sr, Ba).

## Synthesis and crystallization   

The title phosphate, SrCo_2_Fe(PO_4_)_3_, was synthesized in a solid-state reaction by mixing nitrates of strontium, cobalt and iron along with NH_4_H_2_PO_4_, taken in the molar proportions Sr:Co:Fe:P = 1:2:1:3. After a series of heat treatments up to 873 K in a platinum crucible, inter­spersed with grinding, the reaction mixture was heated to the melt (1343 K). The molten product was then cooled to room temperature at 5 K/h. The resulting solid contained brown crystals of a suitable size for X-ray diffraction.

## Refinement   

Crystal data, data collection and structure refinement details are summarized in Table 1 ▸. The highest peak and the deepest hole in the final Fourier map are at 0.63 and 0.68 Å from Sr1 and P2, respectively.

The distinction between cobalt and iron by X-ray diffraction is nearly impossible. Therefore we have examined several crystallographic models during the crystal structure refinements of the title compound. Based on the stoichiometric ratio of 1:2 for iron and cobalt in the starting materials, we assumed the same ratio in the crystal structures with oxidation states of +II for cobalt and and +III for iron. The best model is obtained with Fe1 and Co1 atoms in the Wyckoff positions 4*a* (2/*m*) and 8*g* (2), respectively. This cationic distribution in this model corresponds to the stoichiometry of the expected compound, in addition to the electric neutrality in the structure in reasonable agreement with the final model. 

## Supplementary Material

Crystal structure: contains datablock(s) I. DOI: 10.1107/S2056989016011373/pk2584sup1.cif

Structure factors: contains datablock(s) I. DOI: 10.1107/S2056989016011373/pk2584Isup2.hkl

CCDC reference: 1492743

Additional supporting information: crystallographic information; 3D view; checkCIF report

## Figures and Tables

**Table 1:** Experimental details

Crystal data	
Chemical formula	SrCo_2_Fe(PO_4_)_3_
*M*_r_	546.24
Crystal system, space group	Orthorhombic, *I* *m* *m* *a*
Temperature (K)	296
*a*, *b*, *c* (Å)	10.4097 (2), 13.2714 (3), 6.5481 (2)
*V* (Å^3^)	904.63 (4)
*Z*	4
Radiation type	Mo *K*α
μ (mm^−1^)	11.64
Crystal size (mm)	0.30 × 0.27 × 0.21

Data collection	
Diffractometer	Bruker X8 APEX
Absorption correction	Multi-scan (*SADABS*; Krause *et al.*, 2015 ▸)
*T*_min_, *T*_max_	0.595, 0.747
No. of measured, independent and observed [*I* > 2σ(*I*)] reflections	10008, 1297, 1243
*R*_int_	0.030
(sin θ/λ)_max_ (Å^−1^)	0.858

Refinement	
*R*[*F* ^2^ > 2σ(*F* ^2^)], *wR*(*F* ^2^), *S*	0.017, 0.046, 1.16
No. of reflections	1297
No. of parameters	54
Δρ_max_, Δρ_min_ (e Å^−3^)	1.00, −0.74

Computer programs: *APEX2* and *SAINT* (Bruker, 2009 ▸), *SHELXT2014* (Sheldrick, 2015*a* ▸), *SHELXL2014* (Sheldrick, 2015*b* ▸), *ORTEP-3 for Windows* (Farrugia, 2012 ▸), *DIAMOND* (Brandenburg, 2006 ▸) and *publCIF* (Westrip, 2010 ▸).

## supplementary crystallographic information

### Crystal data

**Table:**

SrCo_2_Fe(PO_4_)_3_	*D*_x_ = 4.011 Mg m^−^^3^
*M**_r_* = 546.24	Mo *K*α radiation, λ = 0.71073 Å
Orthorhombic, *I**m**m**a*	Cell parameters from 1297 reflections
*a* = 10.4097 (2) Å	θ = 3.1–37.6°
*b* = 13.2714 (3) Å	µ = 11.64 mm^−^^1^
*c* = 6.5481 (2) Å	*T* = 296 K
*V* = 904.63 (4) Å^3^	Block, brown
*Z* = 4	0.30 × 0.27 × 0.21 mm
*F*(000) = 1036	

### Data collection

**Table:**

Bruker X8 APEX diffractometer	1297 independent reflections
Radiation source: fine-focus sealed tube	1243 reflections with *I* > 2σ(*I*)
Graphite monochromator	*R*_int_ = 0.030
φ and ω scans	θ_max_ = 37.6°, θ_min_ = 3.1°
Absorption correction: multi-scan (SADABS; Krause *et al.*, 2015)	*h* = −17→17
*T*_min_ = 0.595, *T*_max_ = 0.747	*k* = −22→19
10008 measured reflections	*l* = −11→11

### Refinement

**Table:**

Refinement on *F*^2^	0 restraints
Least-squares matrix: full	*w* = 1/[σ^2^(*F*_o_^2^) + (0.0245*P*)^2^ + 0.761*P*] where *P* = (*F*_o_^2^ + 2*F*_c_^2^)/3
*R*[*F*^2^ > 2σ(*F*^2^)] = 0.017	(Δ/σ)_max_ < 0.001
*wR*(*F*^2^) = 0.046	Δρ_max_ = 1.00 e Å^−^^3^
*S* = 1.16	Δρ_min_ = −0.74 e Å^−^^3^
1297 reflections	Extinction correction: SHELXL2014 (Sheldrick, 2014b), Fc^*^=kFc[1+0.001xFc^2^λ^3^/sin(2θ)]^-1/4^
54 parameters	Extinction coefficient: 0.0131 (4)

### Special details

**Table:**

Geometry. All esds (except the esd in the dihedral angle between two l.s. planes) are estimated using the full covariance matrix. The cell esds are taken into account individually in the estimation of esds in distances, angles and torsion angles; correlations between esds in cell parameters are only used when they are defined by crystal symmetry. An approximate (isotropic) treatment of cell esds is used for estimating esds involving l.s. planes.

### Fractional atomic coordinates and isotropic or equivalent isotropic displacement parameters (Å^2^)

**Table:**

	*x*	*y*	*z*	*U*_iso_*/*U*_eq_	
Sr1	1.0000	0.7500	0.59715 (3)	0.00785 (6)	
Co1	0.7500	0.63284 (2)	0.2500	0.00537 (6)	
Fe1	0.5000	0.5000	0.5000	0.00392 (7)	
P1	1.0000	0.7500	0.09098 (8)	0.00336 (9)	
P2	0.7500	0.42747 (3)	0.2500	0.00388 (7)	
O1	1.0000	0.65633 (9)	−0.04439 (19)	0.00660 (18)	
O2	0.88277 (11)	0.7500	0.23618 (18)	0.00607 (18)	
O3	0.71075 (8)	0.36360 (6)	0.06735 (14)	0.00776 (14)	
O4	0.63833 (7)	0.50376 (6)	0.29533 (14)	0.00600 (13)	

### Atomic displacement parameters (Å^2^)

**Table:**

	*U*^11^	*U*^22^	*U*^33^	*U*^12^	*U*^13^	*U*^23^
Sr1	0.00819 (9)	0.01003 (10)	0.00534 (9)	0.000	0.000	0.000
Co1	0.00533 (9)	0.00376 (10)	0.00704 (10)	0.000	0.00073 (6)	0.000
Fe1	0.00292 (11)	0.00439 (13)	0.00443 (12)	0.000	0.000	0.00015 (9)
P1	0.00344 (18)	0.0029 (2)	0.0038 (2)	0.000	0.000	0.000
P2	0.00410 (14)	0.00365 (17)	0.00388 (14)	0.000	0.00051 (10)	0.000
O1	0.0081 (4)	0.0045 (5)	0.0073 (4)	0.000	0.000	−0.0017 (4)
O2	0.0046 (4)	0.0073 (5)	0.0063 (4)	0.000	0.0020 (3)	0.000
O3	0.0094 (3)	0.0075 (3)	0.0064 (3)	−0.0017 (3)	0.0003 (3)	−0.0023 (2)
O4	0.0050 (3)	0.0057 (3)	0.0074 (3)	0.0013 (2)	0.0021 (2)	0.0006 (2)

### Geometric parameters (Å, º)

**Table:**

Sr1—O1^i^	2.6561 (13)	Fe1—O4	1.9678 (8)
Sr1—O1^ii^	2.6561 (13)	Fe1—O4^xi^	1.9678 (8)
Sr1—O2^iii^	2.6600 (12)	Fe1—O4^xii^	1.9678 (8)
Sr1—O2	2.6600 (12)	Fe1—O4^xiii^	1.9678 (8)
Sr1—O3^iv^	2.6690 (9)	Fe1—O1^iv^	2.0950 (12)
Sr1—O3^v^	2.6690 (9)	Fe1—O1^xiv^	2.0950 (12)
Sr1—O3^vi^	2.6690 (9)	P1—O1^iii^	1.5268 (12)
Sr1—O3^vii^	2.6690 (9)	P1—O1	1.5268 (12)
Co1—O2	2.0824 (8)	P1—O2^iii^	1.5470 (12)
Co1—O2^viii^	2.0824 (8)	P1—O2	1.5470 (12)
Co1—O4^ix^	2.0913 (8)	P2—O3	1.5219 (9)
Co1—O4	2.0914 (8)	P2—O3^ix^	1.5219 (9)
Co1—O3^x^	2.1183 (9)	P2—O4	1.5698 (8)
Co1—O3^iv^	2.1183 (9)	P2—O4^ix^	1.5698 (8)
			
O1^i^—Sr1—O1^ii^	55.81 (5)	O2^viii^—Co1—O3^x^	84.14 (4)
O1^i^—Sr1—O2^iii^	141.74 (2)	O4^ix^—Co1—O3^x^	89.21 (3)
O1^ii^—Sr1—O2^iii^	141.74 (2)	O4—Co1—O3^x^	92.88 (3)
O1^i^—Sr1—O2	141.74 (2)	O2—Co1—O3^iv^	84.14 (4)
O1^ii^—Sr1—O2	141.74 (2)	O2^viii^—Co1—O3^iv^	93.94 (4)
O2^iii^—Sr1—O2	54.61 (5)	O4^ix^—Co1—O3^iv^	92.89 (3)
O1^i^—Sr1—O3^iv^	109.21 (2)	O4—Co1—O3^iv^	89.21 (3)
O1^ii^—Sr1—O3^iv^	78.48 (2)	O3^x^—Co1—O3^iv^	177.44 (5)
O2^iii^—Sr1—O3^iv^	108.19 (3)	O4—Fe1—O4^xi^	94.07 (5)
O2—Sr1—O3^iv^	63.77 (3)	O4—Fe1—O4^xii^	85.93 (5)
O1^i^—Sr1—O3^v^	78.48 (2)	O4^xi^—Fe1—O4^xii^	180.0
O1^ii^—Sr1—O3^v^	109.21 (2)	O4—Fe1—O4^xiii^	180.0
O2^iii^—Sr1—O3^v^	63.77 (3)	O4^xi^—Fe1—O4^xiii^	85.93 (5)
O2—Sr1—O3^v^	108.19 (3)	O4^xii^—Fe1—O4^xiii^	94.07 (5)
O3^iv^—Sr1—O3^v^	171.61 (4)	O4—Fe1—O1^iv^	86.02 (3)
O1^i^—Sr1—O3^vi^	78.48 (2)	O4^xi^—Fe1—O1^iv^	86.02 (3)
O1^ii^—Sr1—O3^vi^	109.21 (2)	O4^xii^—Fe1—O1^iv^	93.98 (3)
O2^iii^—Sr1—O3^vi^	108.19 (3)	O4^xiii^—Fe1—O1^iv^	93.98 (3)
O2—Sr1—O3^vi^	63.77 (3)	O4—Fe1—O1^xiv^	93.98 (3)
O3^iv^—Sr1—O3^vi^	68.78 (4)	O4^xi^—Fe1—O1^xiv^	93.98 (3)
O3^v^—Sr1—O3^vi^	110.56 (4)	O4^xii^—Fe1—O1^xiv^	86.02 (3)
O1^i^—Sr1—O3^vii^	109.21 (2)	O4^xiii^—Fe1—O1^xiv^	86.02 (3)
O1^ii^—Sr1—O3^vii^	78.48 (2)	O1^iv^—Fe1—O1^xiv^	180.0
O2^iii^—Sr1—O3^vii^	63.77 (3)	O1^iii^—P1—O1	109.01 (10)
O2—Sr1—O3^vii^	108.19 (3)	O1^iii^—P1—O2^iii^	110.91 (3)
O3^iv^—Sr1—O3^vii^	110.56 (4)	O1—P1—O2^iii^	110.91 (3)
O3^v^—Sr1—O3^vii^	68.78 (4)	O1^iii^—P1—O2	110.91 (3)
O3^vi^—Sr1—O3^vii^	171.61 (4)	O1—P1—O2	110.91 (3)
O2—Co1—O2^viii^	83.39 (5)	O2^iii^—P1—O2	104.15 (9)
O2—Co1—O4^ix^	103.68 (3)	O3—P2—O3^ix^	112.30 (7)
O2^viii^—Co1—O4^ix^	170.65 (4)	O3—P2—O4	108.00 (5)
O2—Co1—O4	170.65 (4)	O3^ix^—P2—O4	114.17 (5)
O2^viii^—Co1—O4	103.68 (3)	O3—P2—O4^ix^	114.17 (5)
O4^ix^—Co1—O4	70.01 (4)	O3^ix^—P2—O4^ix^	108.00 (5)
O2—Co1—O3^x^	93.94 (4)	O4—P2—O4^ix^	99.68 (6)

Symmetry codes: (i) −*x*+2, −*y*+3/2, *z*+1; (ii) *x*, *y*, *z*+1; (iii) −*x*+2, −*y*+3/2, *z*; (iv) −*x*+3/2, −*y*+1, *z*+1/2; (v) *x*+1/2, *y*+1/2, *z*+1/2; (vi) −*x*+3/2, *y*+1/2, *z*+1/2; (vii) *x*+1/2, −*y*+1, *z*+1/2; (viii) −*x*+3/2, −*y*+3/2, −*z*+1/2; (ix) −*x*+3/2, *y*, −*z*+1/2; (x) *x*, −*y*+1, −*z*; (xi) −*x*+1, *y*, *z*; (xii) *x*, −*y*+1, −*z*+1; (xiii) −*x*+1, −*y*+1, −*z*+1; (xiv) *x*−1/2, *y*, −*z*+1/2.
